# Biogenic synthesis of gold nanoparticles using *Argemone mexicana* L. and their cytotoxic and genotoxic effects on human colon cancer cell line (HCT-15)

**DOI:** 10.1186/s43141-020-00113-y

**Published:** 2021-01-14

**Authors:** Kailas D. Datkhile, Satish R. Patil, Pratik P. Durgawale, Madhavi N. Patil, Dilip D. Hinge, Nilam J. Jagdale, Vinit N. Deshmukh, Ashwini L. More

**Affiliations:** Department of Molecular Biology and Genetics, Krishna Institute of Medical Sciences “Deemed to be University”, Taluka-Karad, Dist-Satara, Malkapur, Maharashtra Pin-415 539 India

**Keywords:** Apoptosis, *Argemone mexicana*, Cell proliferation, Cytotoxicity, Gold nanoparticles

## Abstract

**Background:**

Nanomedicine has evolved as precision medicine in novel therapeutic approach of cancer management. The present study investigated the efficacy of biogenic gold nanoparticles synthesized using *Argemone mexicana* L. aqueous extract (AM-AuNPs) against the human colon cancer cell line, HCT-15.

**Results:**

Biosynthesis of AM-AuNPs was determined by ultraviolet-visible spectroscopy and further characterized by transmission electron microscopy, X-ray diffraction, and Fourier transition infrared spectroscopy analysis. The cytotoxic activity of AM-AuNPs was assessed by the 3-(4, 5-dimethylthiazol-2-yl)-2, 5-diphenyltetrazolium bromide assay, whereas genotoxicity was evaluated by the DNA fragmentation assay. The expression of apoptosis regulatory genes such as p53 and caspase-3 was explored through semi-quantitative reverse transcriptase polymerase chain reaction (RT-PCR) and western blotting to evidence apoptotic cell death in HCT-15 cells. Biogenic AM-AuNPs inhibited cell proliferation in HCT-15 cell line with a half maximal inhibitory concentration (IC_50_) of 20.53 μg/mL at 24 h and 12.03 μg/mL at 48 h of exposure. The altered cell morphology and increased apoptosis due to AM-AuNPs were also evidenced through nuclear DNA fragmentation and upregulated expression of p53 and caspase-3 in HCT-15 cells.

**Conclusion:**

The AM-AuNPs may exert antiproliferative and genotoxic effects on HCT-15 cells by cell growth suppression and induction of apoptosis mediated by activation of p53 and caspase-3 genes.

## Background

Biogenic nanoparticles have attracted interest in recent years due to their potential in industrial, biomedical, and pharmaceutical applications. Metal nanoparticles are of particular significance in several biomedical applications such as targeted drug delivery, bio-imaging, and photo dynamic therapy [1, 2]. As per the world cancer report, cancer is a leading cause of mortality in both developing and developed countries, with 18.1 million new cases and 9.6 million cancer deaths occurring worldwide in 2018 [3]. The current chemotherapeutic approach toward cancer management is insubstantial because of the associated side effects and multidrug resistance. Therefore, researchers worldwide are attempting alternative strategies such as various nano-formulations as promising nano-weapons for efficacious cancer management [4–7]. Metal nanoparticles are considered in biomedical applications because of their biodistribution, augmented efficacy, and reduced toxicity [8, 9]. Biosynthesis of metal nanoparticles is emerging in nanotechnology for the preparation of various homogenous nanoparticles because of its non-toxicity, biocompatibility, and ecofriendliness. The secondary metabolites of plant extracts used in the green synthesis of nanoparticles may enhance their biological potential. The synthesis and biological properties of silver and other metal nanoparticles are widely studied. Biogenic silver nanoparticles are significantly illustrated for their biological efficacies including antimicrobial and anticancer potential, but the gold nanoparticles remained poorly characterized for their biomedical applications. Gold nanoparticles (AuNPs) are being widely used in biomedicine as they can specifically target diseased areas and interact with cellular biomolecules [10–12]. Some studies reported strong anticancer potential of AuNPs synthesized using *Dunaliella salina* and Dragon fruit plants against breast cancer cell lines [13, 14]. Other studies revealed dose-dependent cytotoxicity activity of gold nanoparticles synthesized using plant extracts against lung and bladder cancer cell lines [15–17]. Several in vitro studies have elaborated the mechanism of the anticancer activity of AuNPs where the nanoparticles enter the cells through the permeability and retention effect or nonspecific receptor-mediated endocytosis. Mechanistically, AuNP-treated cancer cell lines displayed generation of reactive oxygen species leading to the membrane damage via oxidation of proteins and lipids, enhanced mitochondrial activity, and ultimately leading to the death of cancer cells [18, 19]. It was also reported that the AuNPs target the cellular organelleles and induce nuclear DNA damage [20]. Although numerous scientists have explored the anticancer potential of biosynthesized nanoparticles, the effect of AuNPs on colon cancer cells remains unclear. Additionally, the intracellular and molecular level interaction of biogenic AuNPs and the precise mechanism involved in the regulation of cytotoxicity and genotoxicity in response to AuNPs remained poorly understood.

*Argemone mexicana*, a Mexican poppy plant, has diverse biological properties such as free radical scavenging, and antimicrobial and antiparasitic activities. Recently, the antiproliferative effects of the *A. mexicana* plant extract have been reported on various cancer cell lines [21, 22]. In earlier studies, we demonstrated that biogenic silver nanoparticles synthesized using *A. mexicana* can induce cell death through inhibition of cell proliferation and the p53-mediated apoptotic pathway in human cancer cells [23]. However, AuNPs of this plant against colon cancer has yet to be studied. Therefore, the current study explored the possible mechanism involved in the inhibition of cell proliferation and involvement of p53 and caspase-3 in the regulation of the apoptotic pathway in HCT-15 cell lines in response to AuNPs biosynthesized using the aqueous extract of *A. mexicana* plant (AM-AuNPs). AM-AuNPs were characterized by UV-visible spectroscopy followed by characterization of size and shape of nanoparticles suing transmission electron microscopy (TEM), X-ray diffraction (XRD), and Fourier transition infrared spectroscopy (FTIR). AM-AuNPs were tested for their cytotoxic activity using the 3-(4, 5-dimethylthiazol-2yl)-2, 5-diphenyltetrazolium bromide (MTT) assay, whereas the genotoxic effects were determined using the DNA fragmentation assay. The apoptotic features were further explored through the profiling of expression of apoptosis regulatory genes at the mRNA and protein level by using semi-quantitative reverse transcriptase polymerase chain reaction (RT-PCR) and western blotting, respectively.

## Methods

### Preparation of aqueous plant extract for nanoparticle synthesis

Whole *A. mexicana* plants were collected from the arid region of the Western Ghats of south-western Maharashtra, India, and authenticated at the Department of Botany, University of Pune, Maharashtra, India. The plant parts were thoroughly washed with distilled water for removing of adhered dust particles, dried, and then ground into fine powder using mortar and pestle. The aqueous extract was prepared by mixing 100 g of leaf powder in 1000 mL of double distilled water and boiled at 80 ^°^C for 30 min in a flask. After cooling, the aqueous extract was filtered through Whatman filter paper. Thereafter, it was passed through a 0.22-μm filter, and the filtrate was used for AuNP biosynthesis.

### Biosynthesis and purification of AuNPs

The AuNPs were synthesized by adding 100 mL of aqueous plant extract to 900 mL of 1 mM gold (III) chloride trihydrate (HAuCl_4_. 3H_2_O) solution. The preparation was then incubated at 80 ^°^C in the dark and monitored for change in color, indicating synthesis of nanoparticles. The AM-AuNPs were centrifuged at 15,000 rpm for 10 min and washed several times with sterile deionized water to remove unwanted traces of contaminants, followed by redispersion of the pellet in sterile distilled water for further characterizations.

### Characterization of biogenic AuNPs

The reduction of gold chloride solution using the aqueous plant extract was monitored for 30, 60, 120, and 180 min, and the appearance of purple color indicated the formation of AuNPs. The primary characterization of the biosynthesized AM-AuNPs was performed using UV-visible spectroscopy by measuring the UV-visible spectrum of the reaction mixture at 200–800 nm wavelength by sampling the aliquots withdrawn from the reaction mixture at different time intervals. The AM-AuNPs were further characterized through TEM, XRD, and FTIR analyses at the Sophisticated Analytical Instrument Facility, Sophisticated Test and Instrumentation Center, Cochin University, Kerala.

### In vitro evaluation of cytotoxicity properties of AM-AuNPs

The HCT-15 cell line obtained from the National Centre for Cell Sciences, Pune, India, was grown in a T-25 flask containing RPMI-1640 medium supplemented with 10% fetal bovine serum (FBS) and 100 U/mL/100 μg/mL penicillin-streptomycin. The cells were maintained at 5% CO_2_, 95% humidity, and 37 ^°^C temperature. The in vitro effect of AM-AuNPs on the cell proliferation of HCT-15 cells was determined by the MTT colorimetric assay. Approximately 1 × 10^4^ cells were seeded in each well of 96-well plate in 200 μL of medium and incubated at 37 °C, 5% CO_2_. After 24 h of incubation, ~ 70% confluent cells were exposed to AM-AuNPs at 5, 10, 20, 25, and 50 μg/mL in the culture medium without FBS and incubated for further 48 h. After completion of 24 h and 48 h treatment, the medium was removed, and the cells were washed with Hanks’ Balanced Salt Solution (HBSS), and cell viability was measured by MTT assay. A total of 10 μL of MTT (5 mg/mL) was added to each well, and the cells were further incubated for another 4 h at 37 °C and 5% CO_2_ atmosphere. After 4 h of incubation, the MTT-containing medium was discarded, and cells were washed with HBSS; thereafter, 200 μL of dimethyl sulfoxide was added to each well to dissolve the formazan crystals. The absorbance of the developed purple color was measured at 560-nm wavelength using the UV-Vis 1800 spectrophotometer (Shimadzu) to determine the percentage inhibition of growth of both treated and untreated cells. The effect of AM-AuNPs on cell proliferation was expressed as percentage (%) growth inhibition, by using the following formula: percentage (%) inhibition = 100 − (A560 nm of treated cells/A560 nm of control cells) × 100%. The half-maximal inhibitory concentration (IC_50_) values with a 95% confidence interval were calculated using the SPSS 11 for Windows software.

### Cell morphology

A total of 1 × 10^6^ HCT-15 cells/well were cultured in 6-well culture plates and incubated at 37 °C in 5% CO_2_ for 24 h. Later, the cells were treated with different concentrations (5, 10, 20, 25, and 50 μg/mL) of AM-AuNPs in culture medium without FBS and further incubated up to 48 h. The cell morphology was then observed under the Primovert phase-contrast microscope at × 20 magnification (Carl Zeiss).

### Evaluation of genotoxic activity of AM-AuNPs

#### DNA fragmentation assay

For analyzing the genotoxic effects of AM-AuNPs, 1 × 10^6^ cells in 6-well plates were treated with 0–50 μg/mL concentrations of AM-AuNPs along with untreated controls and incubated at 37 °C in 5% CO_2_, for 48 h. The cells were harvested by trypsinization with 0.25% trypsin-EDTA for 10 min, and suspended cells were washed once with HBSS by centrifugation. Thereafter, the cells were lysed in 0.3 mL of cell lysis buffer containing 10 mM Tris-HCl, pH 7.5, 1 mM ethylenediaminetetraacetic acid (EDTA), 0.2% triton X-100, 0.5% sodium dodecyl sulfate (SDS). The cell lysate was incubated with 0.5 mg/mL RNase A at 37 °C for 1 h, thereafter, 0.2 mg/mL of proteinase K at 55 °C for 1 h. DNA in the aqueous phase was precipitated by adding 1/10th volume of 5 M sodium chloride and equal volume of isopropanol at − 20 °C. After 1 h incubation, the suspension was centrifuged at 12000 × *g* for 30 min at 4 °C followed by DNA pellet wash by 70% ice cold ethanol and air-dried DNA pellet for 10 min at room temperature. The DNA was resuspended in appropriate volume of T_10_E_1_ buffer (pH 8.0). The DNA was examined on a 1.5% (w/v) low EEO agarose (GeNei) gel containing 1 μg/mL ethidium bromide and subjected to electrophoresis at 80 V for 1–2 h in tris-acetate-EDTA (TAE) buffer along with DNA molecular weight marker. The DNA fragments were visualized by exposing the gels to the UV transilluminator followed by photography in the gel documentation system (BioRad Laboratories, USA).

#### Caspase-3 assay

To examine the apoptosis of HCT-15 cell line, 1 × 10^6^ cells were seeded in 6-well plates and incubated for 24 h at 37 °C in 5% CO_2_. Thereafter, the control and AM-AuNP (12.03 μg/mL)-treated cells were harvested by trypsinization at different time points (0, 24, and 48 h) and washed with ice-cold phosphate-buffered saline. Caspase-3 activity was measured in the cancer cells using a caspase-3 assay kit (Sigma, USA) according to the manufacturer’s instructions. HCT-15 cells were lysed with 100 μL of lysis buffer (50 mM HEPES (pH 7.4), 5 mM CHAPS 5 mM dithiothreitol (DTT) for 30 min at 4 °C). Protein extracts were collected after centrifugation at 12,000 × *g* for 20 min. An equal volume (10 μL) of protein extracts was mixed with the assay buffer (20 mM HEPES (pH 7.4), 0.1% CHAPS, 10 mM DTT, 1 mM EDTA), and incubated with the caspase-3 substrate (acetyl-Asp-Glu-Val-Asp p-nitroanilide (Ac-DEVD-pNA)) and caspase-3 inhibitor (Ac-DEVD-CHO) for 4 h. Then, the absorbance was measured at 405 nm using a double-beam UV-visible spectrophotometer. The assay was also performed with noninduced cells and in the presence of the caspase-3 inhibitor for a comparative analysis.

#### Annexin V-FITC assay

A total of 1 × 10^6^ HCT-15 cell lines were seeded in a 6-well plate and incubated for 24 h at 37 °C in 5% CO_2_, followed by exposure to 5, 10, and 25 μg/mL of AM-AuNPs with incubation periods of 24 and 48 h. After treatment completion, the cells were harvested by trypsinization and washed with 1× HBSS. Thereafter, apoptosis was detected by using the Annexin V-FITC assay kit (Sigma). The cells were resuspended in 1x binding buffer and stained with 5 μL of the Annexin V-FITC conjugate and 10 μL of 100× propidium iodide for 10 min at room temperature in the dark. The cell samples were analyzed using the BD-FACSCelesta flow cytometer (BD-Biosciences) at IISER, Pune.

### Semi-quantitative RT-PCR analysis for apoptotic gene expression

In the present study, total RNA was extracted from the control and AM-AuNP-treated HCT-15 cells at an IC_50_ of 12.03 μg/mL by the Trizol reagent after 24 and 48 h of exposure. RNA quantitation and purity were examined by measuring absorbance of the RNA sample at 260 and 280 nm with the following equation: 1OD at 260 nm = 40 μg. Thereafter, equal amounts of RNA (5 μg) were used for cDNA synthesis in a 20 μL reaction mixture containing 1 μL verso enzyme mix comprising verso reverse transcriptase, 4 μL 5× cDNA synthesis buffer (Thermo Scientific), 5 μg of total RNA, 2 μL of dNTPs (0.5 mM each) (Thermo Scientific), 1 μL of RT enhancer, 1 μL of mixture of random hexamers, and anchored oligo-dT (3:1) (Thermo Scientific). cDNA synthesis was performed by reverse transcription cycling program at 42 °C for 30 min, followed by inactivation at 95 °C for 2 min to remove the contaminating DNA. Thereafter, 2 μL of each cDNA was amplified in a 20 μL PCR reaction mixture containing one unit Taq DNA polymerase, 2 μL 10× PCR buffer, 0.5 μL of dNTP (200 μM each), and 10 picomole of forward primer 5′-actaagcgagcactgcccaa-3′ and reverse primer 5′-atggcgggaggtagactgac-3′) of p53, and forward primer 5′-gtggcattgagacagacagtgg-3′ reverse primer 5′-gccaagaataataaccaggtgc-3′ of caspase-3. Cycling conditions comprised an initial denaturation of 5 min at 94 °C followed by 30 cycles of amplification at 94 °C for 30 s, 50 °C for 30 s, and 72 °C for 30 s, and the final elongation step at 72 °C for 10 min. To control the PCR reaction components and RNA integrity, 2 μL of each cDNA sample was amplified separately for glyceraldehyde 3-phosphate dehydrogenase (GAPDH) specific primers. The electrophoretic separation of amplification products was performed with 2.5% agarose gel in tris-borate-EDTA buffer. The images of ethidium bromide-stained PCR products were captured using Molecular Imager Chemi Doc^TM^ XRS^+^, and the relative density of amplicons was determined using the image analysis software Image Lab^TM^ 3.0 (BioRad Laboratories, USA)

### Western blotting analysis

Western blotting was conducted to detect the expression of apoptotic proteins such as p53 and caspase-3 in HCT-15 cells. A total of 1 × 10^6^ cells 6-well plates were treated with 12.03 μg/mL of AM-AuNPs for 48 h. The cells were harvested with centrifugation at 12,000 rpm for 10 min at 24 h intervals. The cell lysate was prepared in a homogenization buffer (50 mM Tris, 5 mM EDTA, 0.1% Triton-X100, 1 mM PMSF, 0.5 mM DTT, protease inhibitor cocktail). The separation of 50 μg/lane of proteins was performed using 10% SDS-PAGE. They were then transferred onto a nitrocellulose membrane. After blocking in tris-buffered saline, 0.1% Tween-20 solution containing 5% (w/v) casein for 1 h, followed by 1 h incubation with primary antibodies, mouse anti-p53 and rabbit anti-caspase-3, and anti-GAPDH. Thereafter, the membranes were incubated with secondary antibodies (horseradish peroxidase-conjugated rabbit anti-mouse IgG and goat anti-rabbit IgG) for 1 h. Immunoreactive bands were detected using an enhanced chemiluminescence WesternBright^TM^ ECL detection system (Advansta). The western blot images were captured using Molecular Imager Chemi Doc^TM^ XRS^+^ (BioRad Laboratories), and the relative intensity of luminescent bands was determined using the Image Lab^TM^ 3.0 software. The expression of each protein was compared with that of GAPDH to account for potential variations in protein estimation and sample loading.

### Statistical analysis

All the experiments were repeated three times independently (*N* = 3), and means of standard deviation (SD) for each group were calculated. The percentage inhibition of cell growth was reported as mean ± SD of three independent experiments. Student’s *t* test was performed to examine the significant differences between means of expression of p53 and caspase-3 from AM-AuNP treated and control samples both at the mRNA and protein level.

## Results

### Biosynthesis and characterization of AM-AuNPs

**Fig. 1:**
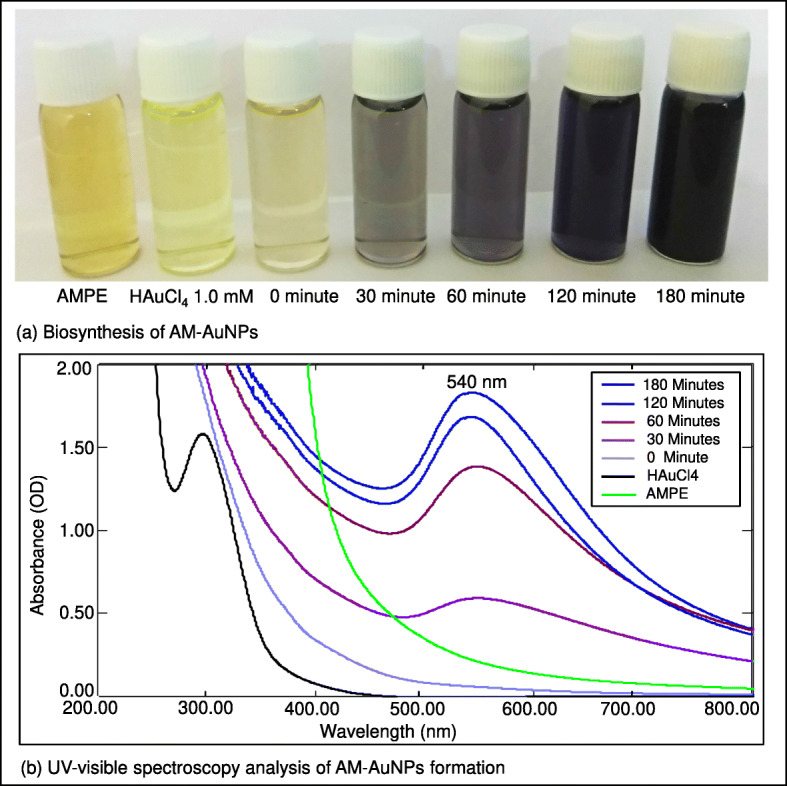
**a** Biosynthesis of gold nanoparticles indicating transformation of color of reaction mixture containing *A. mexicana* plant extract and 1 mM HAuCl4·3H2O solution at various time periods (0–180 min). **b** Time-dependent UV-visible spectrometry analysis of AM-AuNPs formation showing spectra of A*. mexicana* plant extract, HAuCl4·3H2O, and AM-AuNPs. The absorption spectrum of biosynthesized AuNPs exhibited a strong peak at 540 nm

**Fig. 2:**
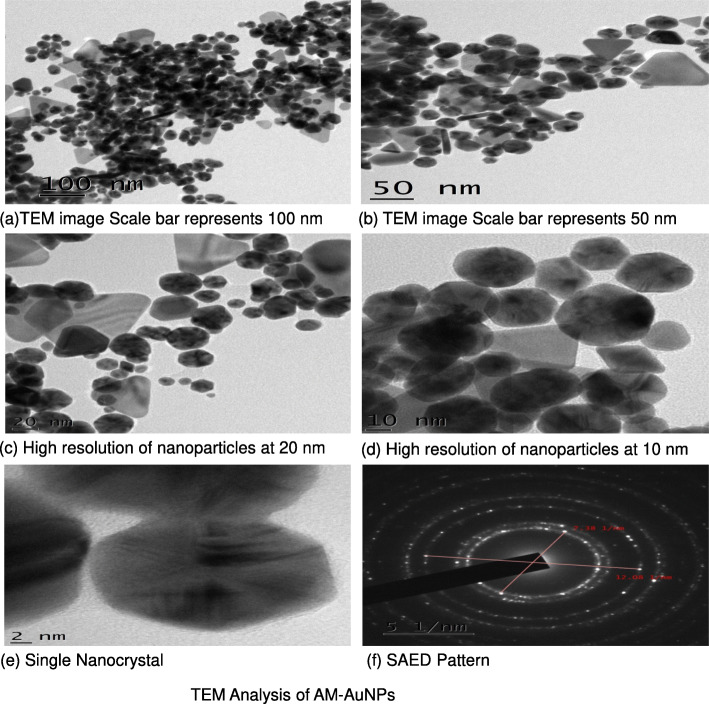
TEM analysis of AM-AuNPs (**a**). Image of AM-AuNPs where scale bar represents 100 nm (**b**). Scale bar represents 50 nm (**c**). High-resolution of nanoparticles at 20 nm (**d**) and 10 nm (**e**). Single nanocrystal (**f**) SAED pattern

**Fig. 3:**
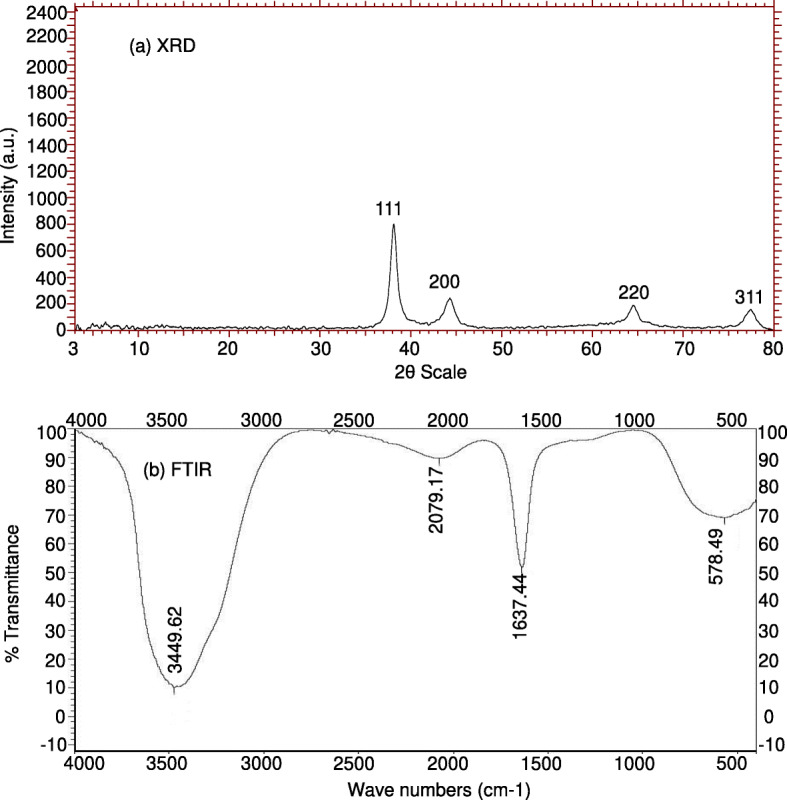
Characterization of AM-AuNPs (**a**) XRD pattern of biogenic gold nanoparticles synthesized using *A. mexicana* plant extract. **b** FTIR spectrum gold nanoparticles synthesized using aqueous extract of *A. mexicana* plant

Reduction of gold chloride solution by the *A. mexicana* aqueous extract determined with the UV-visible absorption spectra at 200–800 nm revealed the formation of AuNPs. The change in the yellow color of the gold chloride solution to dark purple after 3 h of incubation with the *A. mexicana* leaf extract indicated the formation of gold nanoparticles, which may be triggered by phytochemicals present in the plant (Fig. 1a). The time-dependent UV-visible spectra of biosynthesized AM-AuNP solution gave surface plasmon resonance at 540 nm indicating nanoparticle synthesis (Fig. 1b). These results demonstrated that nanoparticle biosynthesis was initiated after 30 min of incubation, and the intensity of peak progressively increased and completed after 3 h of incubation. The TEM results for the characterization of the AM-AuNP, demonstrated mostly hexagonal shape and 20–40-nm-sized AuNPs (Fig. 2a–f). A structural analysis of biosynthesized AuNPs was performed through XRD, and the peaks at 111, 200, 220, and 311 lattice planes clearly confirmed the crystalline structure of AM-AuNPs. The XRD pattern obtained for AM-AuNPs is illustrated in Fig. 3a. The FTIR spectrum of the biosynthesized AuNPs exhibited peaks at 3449 (O–H bond of alcohol/phenol), 2079 (C triple bonds of alkynes), 1637 (C=C of alkynes), and 578 (C–N bond of amines). The FTIR peaks obtained for AM-AuNPs are illustrated in Fig. 3b.

### In vitro cytotoxicity and genotoxicity properties of AM-AuNPs

**Fig. 4:**
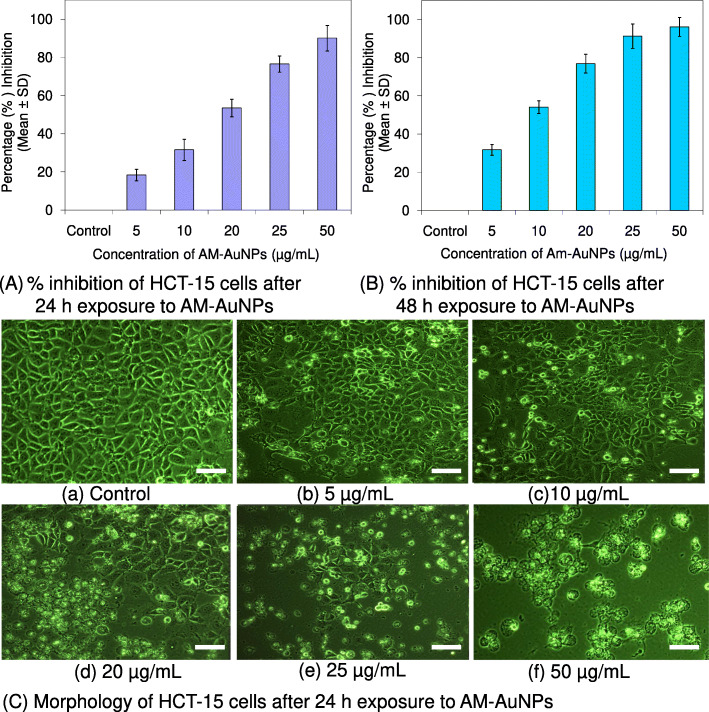
Representative histogram showing percentage growth inhibition of HCT-15 cells after (**A**) 24 h and (**B**) 48 h of exposure to biogenic AM-AuNPs at 5, 10, 20, 25, and 50 μg/mL concentrations. The results represent the means of three independent experiments. Error bars represent the standard deviation of means. (**C**) Morphology of HCT-15 cells after 24 h exposure of AM-AuNPs with different concentrations (a) control; (b) 5 μg/mL; (c) 10 μg/mL; (d) 20 μg/mL; (e) 25 μg/mL; and (f) 50 μg/mL. All images are captured at × 20 magnification with phase contrast microscope. Scale bars represent 100 μm

**Fig. 5:**
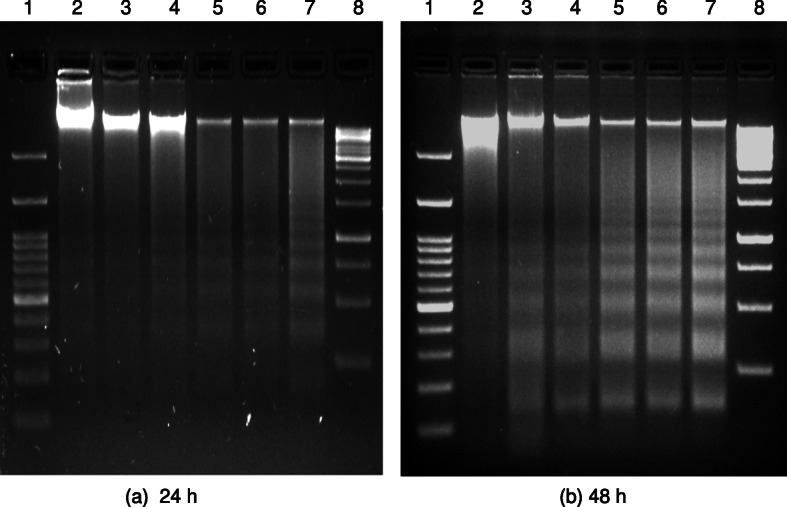
Representative agarose gel images showing DNA fragmentation pattern of HCT-15 cells exposed to different concentrations of AM-AuNPs after different time periods of exposure **a** 24 h and **b** 48 h. In each representative gel, lane 1 is 100 bp ladder; lane 2: DNA of control; followed by lanes 3–7: DNA of AM-AuNP-treated HCT-15 cells exposed to 5, 10, 20, 25, and 50 μg/mL respectively of lane 8 is 1 kb ladder

**Fig. 6:**
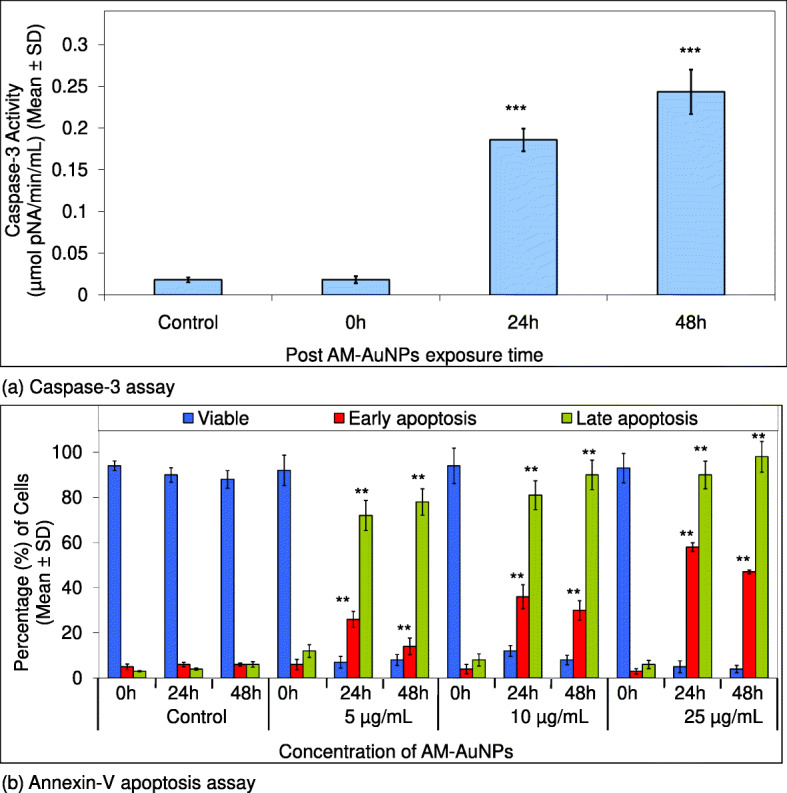
**a** Representative histogram showing caspase-3 activity in HCT-15 cells. Error bars indicate the standard deviation of three independent experiments (*n* = 3). Levels of significance in caspase-3 activity, μmolpNA/min/ml between control and AM-AuNPs exposed cell samples assessed by Student’s *t* test are marked by ***p < 0.0001, which was found to be increased significantly in protein samples obtained from 24 h and 48 h post exposure periods as compared to control cell samples. **b** Histogram showing analysis of Annexin V-FITC-PI staining of AM-AuNP-treated HCT-15 cells for 0, 24, and 48 h. Data presented were expressed as mean ± SD. Significant differences are indicated **p < 0.001 found to be increased significantly in cell samples obtained from 24 and 48 h post exposure to AM-AuNPs as compared to normal control cells

**Fig. 7:**
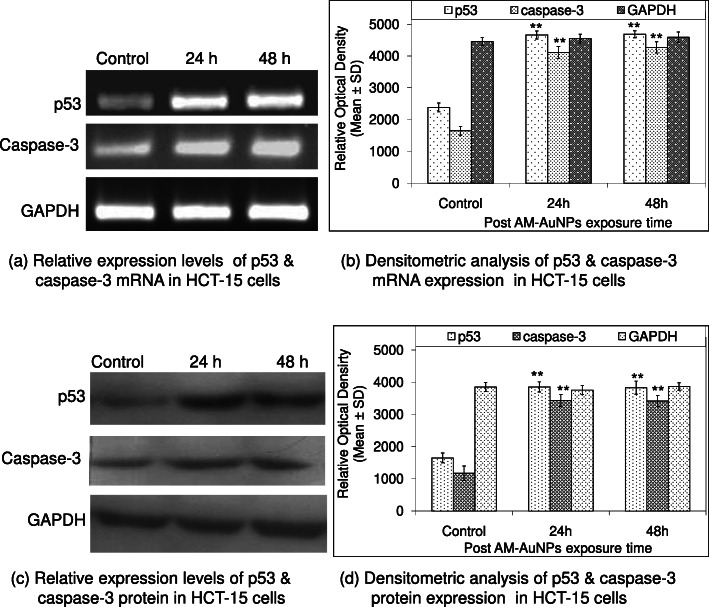
**a** Representative semi-quantitative RT-PCR demonstrates the gene expression changes for p53, caspase-3, and GAPDH from HCT-15 cells exposed to IC_50_ concentration of AM-AuNPs with different time points of exposure 24 and 48 h. Lane 1 is control; lanes 2 and 3 are mRNA levels of AM-AuNPs exposed cell samples after 24 h and 48 h of exposure. **b** Histogram showing densitometry analysis of intrinsic apoptosis-related gene expressions of HCT-15 cells after exposure to AM-AuNPs for 24 and 48 h. Error bars indicate standard deviation of mean for three independent experiments. GAPDH expression was used as loading control. **c** Representative immunoblot showing expression of apoptosis responsive p53 and caspase-3 proteins and GAPDH as internal loading control from HCT-15 cells exposed to IC_50_ concentration of AM-AuNPs for 24 and 48 h. **d** Histogram showing densitometry quantification of apoptotic protein expression. Significant differences assessed by Student’s *t* test are indicated in **p < 0.001, found to be increased significantly in samples obtained from 24 h and 48 h post exposure periods as compared to unexposed control. Error bars indicate standard deviation of mean for three independent experiments

The cytotoxicity activity of biosynthesized AM-AuNPs was explored against HCT-15 cells exposed to 0–50 μg/mL concentrations after 24 h intervals up to 48 h of exposure (Fig. 4a and b). The morphology of HCT-15 cells was altered and exhibited a shrunken appearance because of loss of membrane integrity and cytoplasmic condensation (Fig. 4c) when treated with a higher concentration (20–50 μg/mL) of AM-AuNPs. At 50 μg/mL of AM-AuNPs, maximum growth inhibition (90.10 ± 0.96%) of HCT-15 cells was observed after 24 h of exposure (Fig. 4a) and (96.17 ± 1.15%) after 48 h of exposure (Fig. 4b). The IC_50_ of AM-AuNPs required to inhibit the growth of HCT-15 cells after 24 h of exposure was 20.53 μg/mL whereas the requirement for cells after 48 h of exposure was 12.03 μg/mL. When studying the genotoxic effects of biogenic AuNPs, nuclear fragmentation exhibited clear fragmented DNA from cells exposed to 5–50 μg/mL of AM-AuNPs, whereas the untreated cells did not exhibit any prominent DNA laddering on agarose gels (Fig. 5a and b). DNA fragmentation revealed extensive double-strand breaks in the DNA of HCT-15 cells in response to higher dosages of AM-AuNPs (20–50 μg/mL). Additionally, induction of apoptosis in HCT-15 cells exposed to AM-AuNPs was investigated by the caspase-3 assay. In the cells treated with 12.03 μg/mL of AM-AuNPs for 48 h, caspase-3 activity significantly increased after 24 h and continued till 48 h of exposure compared with the untreated control cells (Fig. 6a). Finally the Annexin-V apoptosis assay was performed to understand the mechanism of death of HCT-15 cells in response to biogenic AM-AuNP exposure. When the cells were treated with increased AM-AuNP concentrations, the AM-AuNPs were more effective with increasing concentration in a time-dependent manner. As the time of incubation at higher concentrations increased from 24 to 48 h, the number of viable cells decreased and the number of cells exhibiting early apoptosis, and thereafter late apoptosis, increased (Fig. 6b). The expression of p53 and caspase-3 genes at the mRNA (Fig. 7a and b) and protein level (Fig. 7c and d) from HCT-15 cells exposed to AM-AuNPs was assessed to study the consequences of biogenic AuNPs on apoptosis. The expression of both p53 and caspase-3 genes/protein was observed to be triggered noticeably in a time-dependent manner.

## Discussion

The present study attempted for the first time to understand the possible mechanism underlying the cytotoxicity and genotoxicity effects of AuNPs synthesized using the *A. mexicana* plant extract. Several studies have reported the plant-mediated biosynthesis of gold nanoparticles, which demonstrated the involvement of phytoconstituents such as alkaloids, flavonoids, and polyphenols in the bioreduction of Au^+^ ions to Au^0^ nanoparticles [24–28]. The antimicrobial and antiparasitic along with cytotoxicity potential have been reported already. Thus, we attempted the biogenic synthesis and exploration of the antiproliferative and genotoxic properties of AM-AuNPs by using colon carcinoma cell line. The biogenic AM-AuNPs exhibited dose-dependent cytotoxic effects with maximum inhibition of growth of HCT-15 cells at 50 μg/mL. Several researchers have reported the cytotoxic effects of biosynthesized AuNPs on different cancer cell lines. The in vitro cytotoxicity of biogenic AuNPs synthesized using Marsilea quadrifolia exhibited an IC_50_ of 45.88 μg/mL toward the PA-1 cell line and 52.01 μg/mL against the A549 cell line [29]. Biologically synthesized gold nanoparticles from *Enicostema axillare* exhibited a strong cytotoxic effect against the MCF-7 cell line [30]. Munawer et al. also evaluated anticancer potential of AuNPs developed using the aqueous extract of *Commiphora wightii* and observed an IC_50_ of 66.11 μg/mL against the breast cancer cell line [31]. Several studies have described the in vitro cytotoxic effects of biogenic AuNPs on several cancer cell lines such as MCF 7, Caco-2, HePG2, and KMST-6 [32]. Similarly, other studies have observed that 100 μg/mL of biogenic AuNPs synthesized using *Nothaphodytes foetida* was required to inhibit 90% growth of HeLa, MCF-7, and HCT-15 cells [33].

Biogenic nanoparticles exhibited cellular apoptosis through a decrease in cell proliferation and an increase in nuclear fragmentation. Our results are concurrent with those of our previous studies when we considered DNA fragmentation-induced apoptosis in HCT-15 cells exposed to biogenic AM-AuNPs. The nuclear DNA fragmentation revealed extensive double-stranded breaks in the DNA of HCT-15 cells when exposed to 20–50 μg/mL AM-AuNPs, whereas the control cells exhibited intact DNA without prominent DNA laddering visible on the agarose gel. Additionally, the apoptotic effects of nanoparticles were confirmed by caspase-3 activation. To examine the status of caspase-3, the caspase-3 activity of HCT-15 cells was observed in response to AM-AuNPs. Its expression increased significantly after 24 and 48 h of exposure. Further, we attempted to understand the mechanism of apoptosis through the Annexin-V assay, where the treatment of cells with an increasing concentration of AM-AuNPs at different incubation periods induced apoptosis in the HCT-15 cell line. This exhibited a shift in the cell population from viable to early apoptosis, and thereafter to the late apoptosis stage. Similar results were reported in gastric cancer and oral squamous cell carcinoma cell lines [34, 35], which demonstrated the status of apoptosis in response to nanoparticles. Inhibition of cell proliferation and induction of apoptosis are biological processes for the regulation of cell survival or death in response to physiological stress conditions. Thus, when HCT-15 cells were treated with an IC_50_ of AM-AuNPs up to 48 h, a significantly increased expression of p53 and caspase-3 at the mRNA and protein level was observed in the AM-AuNP-treated HCT-15 cells in a time-dependent manner, which confirms the induction of apoptosis by AM-AuNPs. Biogenic nanoparticles induced programmed cell death in cancer cells through activation of apoptotic genes [36–39]. Additionally, caspase-3 activity in the AM-AuNP-treated HCT-15 cells was examined to address the potential role of caspase-3 in the apoptotic pathway. The AM-AuNPs increased levels of caspase-3 in the HCT-15 cell line. Thus, the intrinsic apoptotic pathway in HCT-15 cells is mediated through the involvement of p53 and activation of caspase-3 in response to biogenic AM-AuNP exposure.

## Conclusion

The present study addressed the mechanism underlying the cytotoxic and genotoxic effects of biogenic gold nanoparticles synthesized using the aqueous extract of *A. mexicana*. The biosynthesized gold nanoparticles were confirmed through UV-visible spectroscopy, TEM, FTIR, and XRD analyses. The biogenic AM-AuNPs exhibited strong antiproliferative and apoptotic potential against the colon cancer cell line. Our investigation evidently confirmed the intrinsic apoptotic pathway as a core mechanism involved in the cell death of HCT-15 cells exposed to biogenic AM-AuNPs, which was confirmed through semi-quantitative RT-PCR and immunoblotting analysis.

## Data Availability

All data generated and analyzed in this study are included in this manuscript.

## Abbreviations

TEM: Transmission electron microscopy
XRD: X-ray diffraction
FTIR: Fourier transition infrared spectroscopy
MTT: 3-(4, 5-dimethylthiazol-2-yl)-2,5-diphenyltetrazolium bromide
IC50: 50% inhibitory concentration
P53: Tumor suppressor protein
AM-AuNPs: *Argemone mexicana* gold nanoparticles
RT-PCR: Reverse transcriptase–polymerase chain reaction
